# Protective Effects of 7S,15R-Dihydroxy-16S,17S-Epoxy-Docosapentaenoic Acid (diHEP-DPA) against Blue Light-Induced Retinal Damages in A2E-Laden ARPE-19 Cells

**DOI:** 10.3390/antiox13080982

**Published:** 2024-08-13

**Authors:** Seung-Yub Song, Dae-Hun Park, Sung-Ho Lee, Han-Kyu Lim, Jin-Woo Park, Jeong-Woo Seo, Seung-Sik Cho

**Affiliations:** 1 Department of Pharmacy, College of Pharmacy, Mokpo National University, Muan 58554, Jeonnam, Republic of Korea; tgb1007@naver.com (S.-Y.S.); tjdgh0730@naver.com (S.-H.L.); jwpark@mnu.ac.kr (J.-W.P.); 2 Biomedicine, Health & Life Convergence Sciences, BK21 Four, College of Pharmacy, Mokpo National University, Muan 58554, Jeonnam, Republic of Korea; limhk@mokpo.ac.kr; 3 College of Oriental Medicine, Dongshin University, Naju-si 58245, Jeonnam, Republic of Korea; dhj1221@hanmail.net; 4 Department of Marine and Fisheries Resources, Mokpo National University, Muan 58554, Jeonnam, Republic of Korea; 5 Microbial Biotechnology Research Center, Korea Research Institute of Bioscience and Biotechnology (KRIBB), Jeongeup-si 56212, Jeollabuk-do, Republic of Korea; jwseo@kribb.re.kr

**Keywords:** docosahexaenoic acid, diHEP-DPA, blue light, eye health, ARPE-19

## Abstract

The purpose of this study was to investigate the protective effects of 7S,15R-dihydroxy-16S,17S-epoxy-docosapentaenoic acid (diHEP-DPA) in retinal pigment epithelial (RPE) cell damage. ARPE-19 cells, a human RPE cell line, were cultured with diHEP-DPA and Bis-retinoid N-retinyl-N-retinylidene ethanolamine (A2E), followed by exposure to BL. Cell viability and cell death rates were determined. Western blotting was performed to determine changes in apoptotic factors, mitogen-activated protein kinase (MAPK) family proteins, inflammatory proteins, and oxidative and carbonyl stresses. The levels of pro-inflammatory cytokines in the culture medium supernatants were also measured. Exposure to A2E and BL increased the ARPE-19 cell death rate, which was alleviated by diHEP-DPA in a concentration-dependent manner. A2E and BL treatments induced apoptosis in ARPE-19 cells, which was also alleviated by diHEP-DPA. Analysis of the relationship with MAPK proteins revealed that the expression of p-JNK and p-P38 increased after A2E and BL treatments and decreased with exposure to diHEP-DPA in a concentration-dependent manner. DiHEP-DPA also affected the inflammatory response by suppressing the expression of inflammatory proteins and the production of pro-inflammatory cytokines. Furthermore, it was shown that diHEP-DPA regulated the proteins related to oxidative and carbonyl stresses. Taken together, our results provide evidence that diHEP-DPA can inhibit cell damage caused by A2E and BL exposure at the cellular level by controlling various pathways involved in apoptosis and inflammatory responses.

## 1. Introduction

Docosahexaenoic acid (DHA) is the major omega-3 long-chain polyunsaturated fatty acid (n-3 PUFA) found in the human brain and retina in eyes [1]. In response to pathogen invasion or tissue injury, polyunsaturated fatty acids such as DHA are released locally from membrane phospholipids or delivered to sites of inflammation by tissue oedema for subsequent conversion to specialized mediators by cells in the exudates [2]. DHA is closely related to fetal development [3], prevents preterm birth and cardiovascular disease [4], and improves cognitive function and eye health in adults and older adults [5].

In particular, DHA is an important factor in the development of fetuses and infants, but it has recently been reported that consuming n-3 PUFA or fish improves eye health in the elderly [5]. In particular, with regard to AMD, Christen et al. and Seddon et al. reported that high intake of n-3 PUFA as well as fish intake are effective for early and late AMD [6,7].

DHA is known to be most abundant in fish oil, with a DHA content of approximately 12% [1]. Highly concentrated oil produced from fish oil is sold as a medicinal or functional food. Recently, research on the synthesis of DHA derivatives through enzymatic reactions has been conducted, with a focus on lipid autacoids such as specialized pro-resolving mediators (SPM). SPMs are composed of lipoxins, E-series, and D-series resolvins, protectins, and maresins. Individual members of the SPM family serve as agonists at cognate receptors to induce cell-type specific responses. E-type resolvin can be synthesized from eicosapentanoic acid, and the representative substances are RvE1 and RvE2. In docosahexanoic acid, D-type resolvin, protectin, and maresin are derived. RvD1 is the most common substance in D-type resolvin. Protectin D1 and NPD1 are representative protectins. Mar1 is a typical SPM for Maresins [8].

SPMs are enzymatically extracted from essential fatty acids and play important roles in tissue inflammation. In addition, lipid mediators are part of a large family of pro-catalytic molecules, including proteins and gases that suppress inflammation.

Inflammatory responses are regulated in vivo by several pro-inflammatory mediators, including various lipid mediators (e.g., prostaglandins and leukotrienes), cytokines, and chemokines. These events have overlapping and distinct functions, ultimately leading to increased vascular permeability and the regulation of leukocyte migration. This leads to various dysfunctions, including inflammation, erythema, and tumors, which are the main signs of tissue inflammation [9].

Recently, SPMs have received sustained attention as anti-inflammatory factors that are produced during acute inflammatory responses. Many researchers have attempted to synthesize new SPMs [9,10,11]. intracellular inflammatory responses, granulocytes cause apoptosis in tissues. Subsequently, apoptotic neutrophils induce the conversion of macrophages into anti-inflammatory macrophages [12]. then produced by efferocytosis, contributing to fever, inflammation, and pain relief [13].

In response to pathogen invasion or tissue damage, polyunsaturated fatty acids are released locally from membrane phospholipids or delivered to the site of inflammation by tissue edema and are converted into special mediators by cells in the exudate [2]. Arachidonic acid (C20:4n-6) produces eicosanoids (prostaglandins and cysteinyl leukotrienes) that are involved in metabolism and can direct peripheral blood neutrophils to infected areas. Prostaglandin E2 (PGE2) and PGI2 regulate blood flow, while leukotriene C4 (LTC4) and LTD4 regulate vascular permeability. Neutrophils can move to the site of inflammation via the chemotaxis of LTB4 [14,15]. Early in the acute inflammatory response, pro-resolving mediator biosynthesis is initiated through a lipid mediator class switch, in which arachidonic acid metabolism switches from leukotriene production to lipoxin production [16].

SPMs can be extracted from essential fatty acids, including arachidonic acid, eicosapentaenoic acid (EPA; C20:5n-3), and docosahexaenoic acid (DHA; C22:6n-3), using lipoxygenase (LOX). SPMs are stereoselective, and the structural analysis of most SPMs is known [17].

Lipoxins are well studied SPMs. Lipoxins can be produced by intercellular biosynthesis via various pathways [18]. The first pathway is synthesized by 5-LOX from leukocytes and 12-LOX from platelets in the vascular system, whereas the second pathway involves epithelial cells, eosinophils, and monocytes. One example is the pathway through which SPMs are produced from arachidonic acid by 15-LOX or 5-LOX produced by leukocytes [19,20]. In addition to lipoxins, SPMs are converted from omega-3 fatty acids in resolving exudates; representative SPMs include resolvin, protectin, and maresin [10].

The two major resolvins derived from DHA are the D family (RvD1–RvD6) and AT isomers (AT-RvD1–RvD6) [21]. D-series resolvins are produced enzymatically by 15-LOX-mediated conversion of DHA to 17(S)-hydroperoxyDHA (17(S)-HpDHA) and subsequent conversion by 5-LOX [22].

Recent studies have reported that SPMs such as 17(S)-hydroxy-DHA and protectin D1 are produced in the human respiratory tract because DHA is abundant in the respiratory mucosa of healthy individuals [23,24].

SPMs have been reported to control tissue damage by controlling inflammation at extremely low concentrations in the picogram-to-nanogram range. In particular, fatty acid-derived substances such as SPMs are known to be involved in resolution responses along with annexin A1 [25], TGFβ, interleukin-10 [26], microRNA [27], and carbon monoxide [28].

Based on the role of these in vivo SPMs in restoring normal cell damage through strong autacoid behavior at low concentrations, this study aimed to develop SPM derivatives that could be administered orally and externally.

SPMs exert immunomodulatory effects on various cells at the pico/nanogram level. The main cell types of SPMs include neutrophils [9], monocytes, macrophages [11], natural killer cells [29], Tregs [30], and bronchial epithelial cells [31].

To date, research has mainly been performed on SPMs in inflammation and immune-related cells, but there has been no research on new SPMs in eye-related cells (retinal pigment epithelial [RPE] cells), which our research team targeted in this study.

Recently, it has been reported that excessive viewing of video devices such as smartphones increases the risk of myopia, strabismus, dry eye syndrome, and macular degeneration [32]. In particular, there is a risk of continuous exposure to blue light (BL), which is known to be one of the causes of age-related macular degeneration [33]. This is being emphasized. emitted from imaging devices is a type of visible light with a short wavelength of 380–500 nm (nanometers). The shorter the wavelength, the greater the energy, and when accumulated, it causes substantial damage to retinal cells [34]. Due to their high energy, short wavelengths can increase free radicals in the body, damaging the DNA of cells and are known to be one of the risk factors for uveal melanoma.

Recently, long-term exposure to BL was shown to cause excessive oxidative stress in dry age-related macular degeneration (AMD). In a mouse in vivo long-term exposure model, BL weakened the retinal layer, caused apoptosis of retinal cells, and intensified damage to the mitochondria. Particularly, mitochondrial function has been reported to deteriorate [35].

Previously, we obtained a new SPM derivative, 7S,15R-Dihydroxy-16S,17S-epoxy-docosapentaenoic acid (diHEP-DPA), through enzyme synthesis and reported that it showed anti-cancer [36], anti-ulcerative [37], and anti-inflammatory [38] effects via oral administration in vivo.

While previous studies examined the possibility of developing diHEP-DPA as an oral preparation, in the present study, considering the hydrophobicity of diHEP-DPA and its high activity at concentrations below micromolar levels, basic experiments were conducted to develop an ophthalmic drug.

In the present study, we analyze the mechanism of action of diHEP-DPA at the cellular level. The results of our study provide evidence for the development of diHEP-DPA as an effective ocular drug for the treatment of macular degeneration. Our findings provide guidance for future in vivo tests, administration routes, and mechanism studies in tissue.

## 2. Materials and Methods

### 2.1. Cell Culture

Human RPE cells (ARPE-19 cells) were purchased from the American Type Culture Collection (ATCC, Manassas, VA, USA). The ARPE-19 cells were maintained in Dulbecco’s Modified Eagle’s Medium (DMEM, Gibco, Paisley, UK). The medium was supplemented with 10% fetal bovine serum (FBS, Gibco) and 1% penicillin/streptomycin (Gibco) at 37 °C and kept in an atmosphere containing 5% CO_2_.

### 2.2. Cell Viability Assay

Cell viability was measured by the 3-(4,5-dimethylthiazol-2-yl)-2,5-diphenyltetrazolium bromide (MTT, Sigma-Aldrich, Saint Louis, MO, USA) method. diHEP-DPA was obtained from DHA through an enzymatic reaction using cyanobacterial lipoxygenase and purified (purity > 98%) as previously described [39]. ARPE-19 cells were seeded in a 96 well-plate density of 5 × 10^3^ cells/well containing 100 μL of the culture medium. After 24-h incubation, the cells were continuously treated with lutein (Sigma-Aldrich, 10 μM), diHEP-DPA (1, 5, or 10 μM) for 24 h. After 10 μL of MTT solution (5 mg/mL) were added to each well, the plates were further incubated for 2 h. After the medium was removed, formazan crystals were dissolved with 100 μL of dimethyl sulfoxide (DMSO). Absorbance was then measured at a wavelength of 570 nm using a Multiskan SkyHigh Microplate Spectrophotometer (Thermo Fisher Scientific, Waltham, MA, USA). Afterwards, ARPE-19 cells (2 × 10^3^ cells/well) were treated with lutein (Sigma-Aldrich, 10 μM) and diHEP-DPA (1, 5, or 10 μM) for 24 h. After A2E (10 μM) treatment, the cells were exposed to BL (20 mW/cm^2^) for 15 min. After 24 h, an MTT assay was then conducted to evaluate the inhibition of A2E- and BL-induced ARPE-19 cells.

### 2.3. Fluorescence Activated Cell Sorting (FACS) Assay

ARPE-19 cells were seeded in a 6-well plate (2 × 10^5^ cells/well) for 24 h. Thereafter, different concentrations of diHEP-DPA (1, 5, or 10 μM) and lutein (Sigma-Aldrich, 10 μM) were added for 24 h. After A2E (10 μM) treatment, exposure to BL (20 mW/cm^2^) for 15 min was performed. After 24 h, the cells were washed with PBS (Lonza, Walkersville, MD, USA), harvested using trypsin, and centrifuged. The cells were then resuspended in binding buffer (400 μL) and incubated in Alexa Fluor 488 annexin V (5 μL) and PI (1 mg/mL, 1 μL) for 15 min under room temperature and darkness. Cell sorting analysis of the collected cells was performed using the Guava Easycyte reagent (Millipore, Burlington, MA, USA).

### 2.4. TUNEL Assay

A Click-iT^TM^ Plus TUNEL assay kit (Invitrogen, Carlsbad, CA, USA) was used, and all assays were conducted according to the manufacturer’s guidelines. ARPE-19 cells were seeded in a 4-chamber with 1 × 10^4^ cells/well. After 24 h, the medium was removed and treated with diHEP-DPA (1, 5, or 10 μM) and lutein (10 μM, Sigma-Aldrich, Saint Louis, MO, USA). After 24 h and A2E (10 μM) treatment, the cells were exposed to BL (20 mW/cm^2^) for 15 min. The medium was then removed, and the cells were washed with PBS (Lonza, Walkersville, MD, USA) and fixed with 4% formaldehyde (Daejung, Siheung-si, Gyeonggi-do, Republic of Korea) in PBS for 15 min. Thereafter, 0.25% Triton X-100 (Sigma-Aldrich, Saint Louis, MO, USA) was added to fixed cells in PBS for 10 min. The cells were then incubated in 50 liters of TdT reaction solution for 60 min at 37 °C. The nucleus was then stained with DAPI (Thermo Fisher, Waltham, MA, USA), and images were acquired using a K1-Fluo confocal microscope (excitation and emission 495/519 nm Nanoscope Systems, Daejeon, Republic of Korea).

### 2.5. Western Blot

ARPE-19 cells were seeded in 100 a ϕ dish with 4 × 10^5^ cells/well. After 24 h, the medium was removed and treated with diHEP-DPA (1, 5, or 10 μM) and lutein (Sigma-Aldrich, 10 μM). After 24 h, the supernatants were aspirated and treated with A2E (10 μM) and BL (20 mW/cm^2^) for 15 min. The cells were collected using a protease inhibitor cocktail (Thermo Fisher Scientific) and RIPA-based lysis buffer (Thermo Fisher Scientific) and centrifuged for 20 min. Protein concentrations were determined using a bicinchoninic acid protein assay kit (Thermo Fisher Scientific). The proteins were separated by SDS-PAGE, and electric transfer was conducted with a PVDF membrane for 3 h at 100 V. The membrane was incubated in 5% skin milk blocking for 2 h at 4 °C. Primary antibodies were incubated overnight at 4 °C. Primary antibodies were used for Bcl-xL (Invitrogen, Carlsbad, CA, USA), Bcl-2 (Invitrogen), Bad (Santa crusz biotechnology, Dallas, TX, USA), Bim (Santa crusz biotechnology), *p*-JNK (Cell signaling technology, 9102, Danvers, MA, USA), JNK (Cell signaling technology), P38 (Cell signaling technology), *p*-NF-κB (Thermo Fisher Scientific Inc.), NF-κB (Invitrogen), COX-2 (Abcam, Cambridge, UK), PGE_2_ (Bioss, Woburn, MA, USA), Keap1 (Invitrogen), Nrf2 (Invitrogen), SOD1 (Invitrogen), iNOS (Invitrogen), 4-HNE (Abcam), and GADPH (Invitrogen). Primary antibodies were used at 100-fold (Bad, Bim), 1000-fold (Bcl-xL, Bcl-2, p-JNK, JNK, p-P38, P38, p-NF-κB, NF-κB, COX-2, PGE2, Keap1, Nrf2, SOD1, iNOS, and 4-HNE), and 5000-fold (GAPDH) dilutions. All secondary antibodies were used at 5000-fold dilutions. The membrane was washed three times with Tris-buffered saline containing Tween (TBST) for 15 min. The membranes were incubated with secondary antibodies for 2 h at 4 °C. The secondary antibodies used were goat anti-rabbit IgG (Jackson ImmunoResearch, 111-035-003, West Grove, PA, USA) and goat anti-mouse IgG (Jackson ImmunoResearch, 115-035-003). The protein bands were detected using an enhanced chemiluminescence kit (Thermo Fisher Scientific and Davinch-Western^TM^, Davinch-K, Seoul, Republic of Korea).

### 2.6. ELISA Analysis

Tumor necrosis factor alpha (TNF-α), interleukin 1-beta (IL-1β), and interleukin-6 (IL-6) concentrations were measured using the Human ELISA kit (Invitrogen, BD Biosciences, Franklin Lakes, NJ, USA). Capture antibodies were incubated overnight at 4 °C. After The plate was washed three times with 0.05% Tween-20 in PBS, and standards and samples (100 μL) were incubated for 2 h at 4 °C. Thereafter, the plate was removed, and the standard and sample solutions were incubated (100 μL/well) for 1 h at room temperature for the detection of antibodies. After the plate was added, stop solution (50 μL/well) was measured at 450 nm with a microplate reader (Perkin Elmer, Waltham, MA, USA).

### 2.7. Immunofluorescence (IF) Analysis

*p*-NF-κB and COX-2, Nrf2, and Keap1 expression levels were measured using immunofluorescence. ARPE-19 cells were seeded in 4-chamber with 1 x 10^4^ cells/well. After 24 h, the medium was removed and treated with diHEP-DPA (1, 5, or 10 μM) and lutein (Sigma-Aldrich, 10 μM). After 24 h, the cells were treated with A2E (10 μM) and exposed to BL (20 mW/cm^2^) for 15 min. The medium was then removed, and the cells were washed with PBS (Lonza, Walkersville, MD, USA) and fixed with 4% formaldehyde (Daejung, Siheung-si, Gyeonggi-do, Republic of Korea) in PBS for 15 min. Then, 0.25% Triton X-100 (Sigma-Aldrich, Saint Louis, MO, USA) was added to the fixed cells in PBS for 5 min. The cells were then blocked with 1% Bovine Serum Albumin (BSA) for 1 h, and primary antibodies were incubated overnight at 4 °C. Thereafter, the cells were washed thrice with PBS (Lonza) for 5 min. Cells were incubated with secondary antibodies for 2 h under 4 °C and darkness. The nucleus was then stained with DAPI (Thermo Fisher). *p*-NF-κB (Thermo Fisher Scientific Inc.), COX-2 (Abcam), Nrf2 (Invitrogen), Keap1 (Invitrogen), Alexa Fluor 488-conjugated anti-rabbit IgG (493/518mm, A3273, Invitrogen), and Alexa Fluor 555-conjugated anti-goat IgG (553/568nm, A32816, Invitrogen) were used. A K1-Fluo confocal microscope (Nanoscope Systems) was used for image acquisition and fluorescence intensity analysis.

### 2.8. Statistical Analysis

The results are expressed as mean ± standard deviation (SD). Group differences were evaluated using one-way analysis of variance (ANOVA), followed by Dunnett’s multiple comparison test. Statistical significance was set at *p* < 0.05.

## 3. Results and Discussion

### 3.1. diHEP-DP- Inhibited Cell Death Caused by A2E Treatment and BL Exposure

**Figure 1:**
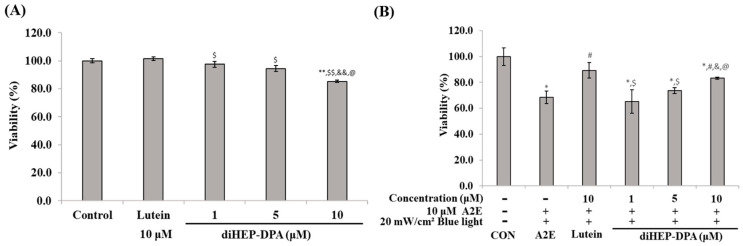
Effects of diHEP-DPA on cell viability. (**A**) Viability of ARPE-19 cells treated with lutein (10 µM) and diHEP-DPA (1 to 10 µM) for 24 h; (**B**) diHEP-DPA prevented A2E and blue light-induced cell death. The values are expressed as the mean ± S.D. (*n* = 3) of three individual experiments. “*” *p* < 0.05 vs. CON; “**” *p* < 0.001 vs. CON; “#” *p* < 0.05 vs. A2E; “$” *p* < 0.05 vs. Lutein; “$$” *p* < 0.001 vs. Lutein; “&” *p* < 0.05 vs. 1 µM; “&&” *p* < 0.001 vs. 1 µM; “@” *p* < 0.05 vs. 5 µM.

As shown in Figure 1A, lutein and diHEP-DPA are considered safe at concentrations within 10 μM, so future experiments were performed within the concentration range of 10 μM. A2E and BL-induced cytotoxicity was reduced in a concentration-dependent manner. When treated with A2E and BL, the cell survival rate was about 70%; Lutein showed a survival rate of 89% at a concentration of 10 μM, and diHEP-DPA showed a survival rate of 83% at a concentration of 10 μM (Figure 1B).

### 3.2. diHEP-DPA Regulated the Apoptosis Caused by BL in A2E-Laden ARPE-19 Cells

**Figure 2:**
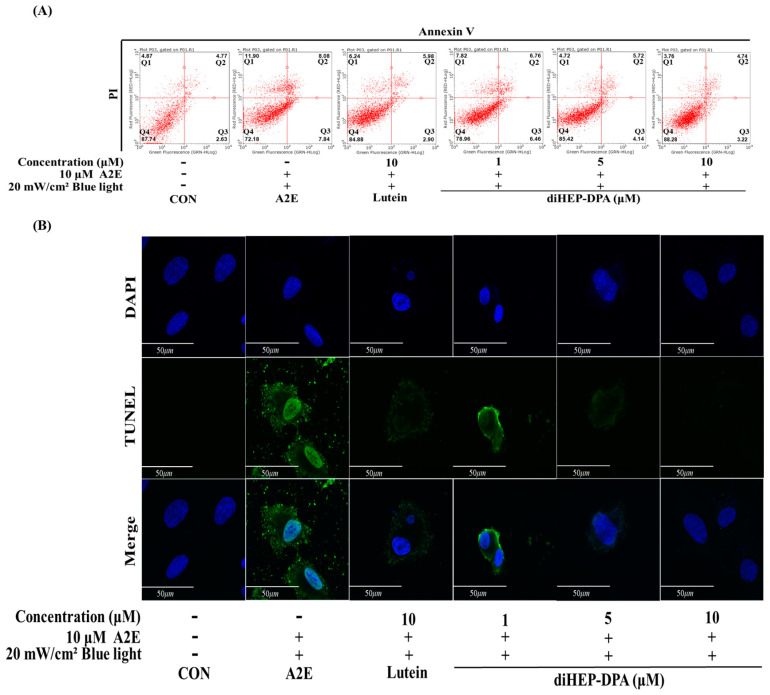
diHEP-DPA decreases apoptosis and necroptosis induced by blue light in A2E-laden retinal pigment epithelium (ARPE-19) cells. (**A**) Representative dot-plots showing the dual parameters used for Annexin V/PI staining and flow cytometry. Q1: Necroptosis cells; Q2: Late apoptosis cells; Q3: Early apoptosis; Q4: Live cells; (**B**) TUNEL results showed that the apoptosis decreased with the increase in DIHEP-DPA concentration. ARPE-19 cells were fixed and probed against α-tubulin (green). The cells were counterstained with DAPI (blue) and visualized by confocal microscopy. Scale bor, 50 µm. Magnifiaction ×400.

As shown in Figure 2, when cells were treated with A2E and BL simultaneously, apoptotic cells increased to 13%, but the lutein (10 µM) treatment group showed a 10% reduction effect. diHEP-DPA reduced apoptotic cells in a concentration-dependent manner to 8% at a concentration of 10 µM. Figure 2B shows that diHEP-DPA reduced A2E- and BL-induced apoptosis in a concentration-dependent manner.

**Figure 3:**
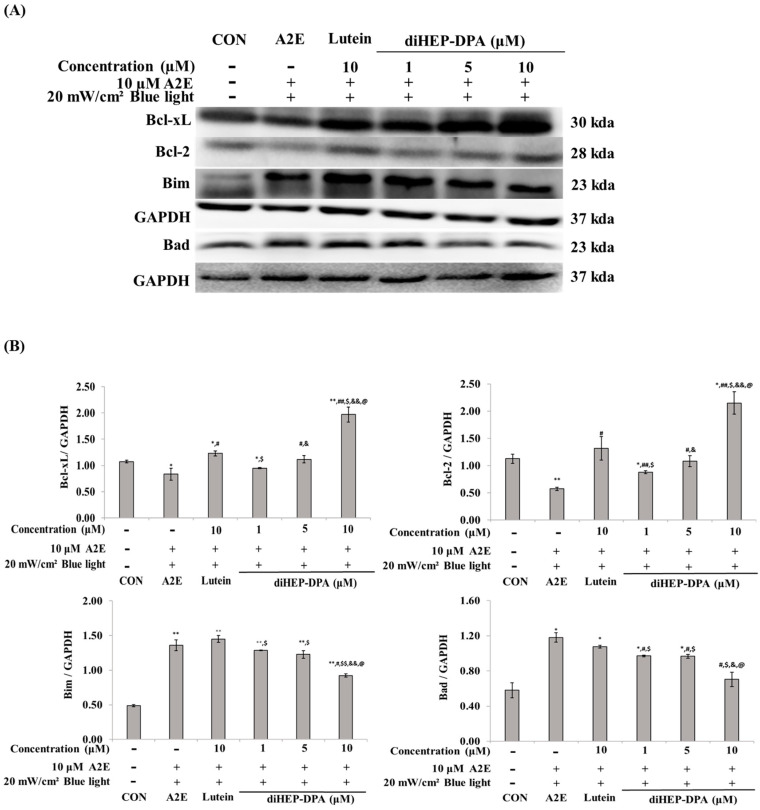
Western blot analysis of Bcl-family proteins induced by blue light in A2E-laden retinal pigment epithelium (ARPE-19) cells. (**A**) Western blot analysis indicating the expression of Bcl-xL, Bcl-2, Bax, Bad, Bim, and GAPDH. (**B**) Quantifications were approximated using densitometry (Image J software version 1.8.0), and results were normalized to GAPDH. The values are expressed as the mean ± S.D. (n = 3) of three individual experiments. “*” *p* < 0.05 vs. CON; “**” *p* < 0.001 vs. CON; “#” *p* < 0.05 vs. A2E; “##” *p* < 0.001 vs. A2E; “$” *p* < 0.05 vs. Lutein; “$$” *p* < 0.001 vs. Lutein; “&” *p* < 0.05 vs. 1 µM; “&&” *p* < 0.001 vs. 1 µM; “@” *p* < 0.05 vs. 5 µM.

Figure 3 shows the expression regulation pattern for apoptosis-related biomarkers. When A2E and BL were treated simultaneously, the expression of Bcl-xL and BCl-2 decreased, but the lutein 10 µM treatment group and the diHEP-DPA 5 µM treatment group were found to be restored to almost the same level as the control. In the case of Bad and Bim, lutein could not reduce the increased expression by A2E and BL, but diHEP-DPA decreased the expression level in a concentration-dependent manner. 

### 3.3. diHEP-DPA Regulated the JNK and p38 in A2E and BL-Induced Apoptosis

**Figure 4:**
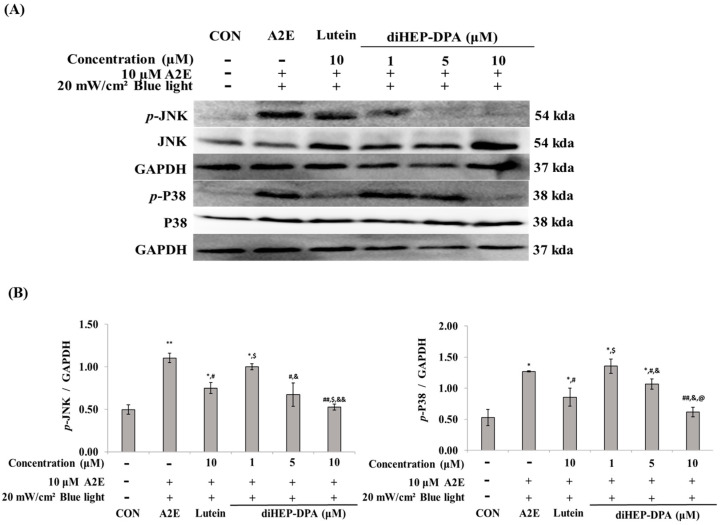
Western blot analysis of mitogen-activated protein kinase (MAPK) family proteins induced by blue light in A2E-laden retinal pigment epithelium (ARPE-19) cells. (**A**) Western blot analysis indicating the expression of *p*-JNK, JNK, *p*-P38, P38, and GAPDH. (**B**) Quantifications were approximated using densitometry (Image J software version 1.8.0), and results were normalized to GAPDH. The values are expressed as the mean ± S.D. (n = 3) of three individual experiments. “*” *p* < 0.05 vs. CON; “**” *p* < 0.001 vs. CON; “#” *p* < 0.05 vs. A2E; “##” *p* < 0.001 vs. A2E; “$” *p* < 0.05 vs. Lutein; “&” *p* < 0.05 vs. 1 µM; “&&” *p* < 0.001 vs. 1 µM; “@” *p* < 0.05 vs. 5 µM.

As shown in Figure 4, by examining the expression of MAPK-related proteins related to apoptosis, we found that the phosphorylation of p38 and JNK increased when A2E and BL were simultaneously administered (Figure 4A), and diHEP-DPA reduced the expression of *p*-p38 and *p*-JNK in a concentration-dependent manner. When compared to the control group (lutein, 10 µM) at the same concentration, diHEP-DPA strongly inhibited the expression of *p*-p38 and *p*-JNK (Figure 4B).

### 3.4. diHEP-DPA Regulated the Inflammatory Response Caused by BL in A2E-Laden ARPE-19 Cells

**Figure 5:**
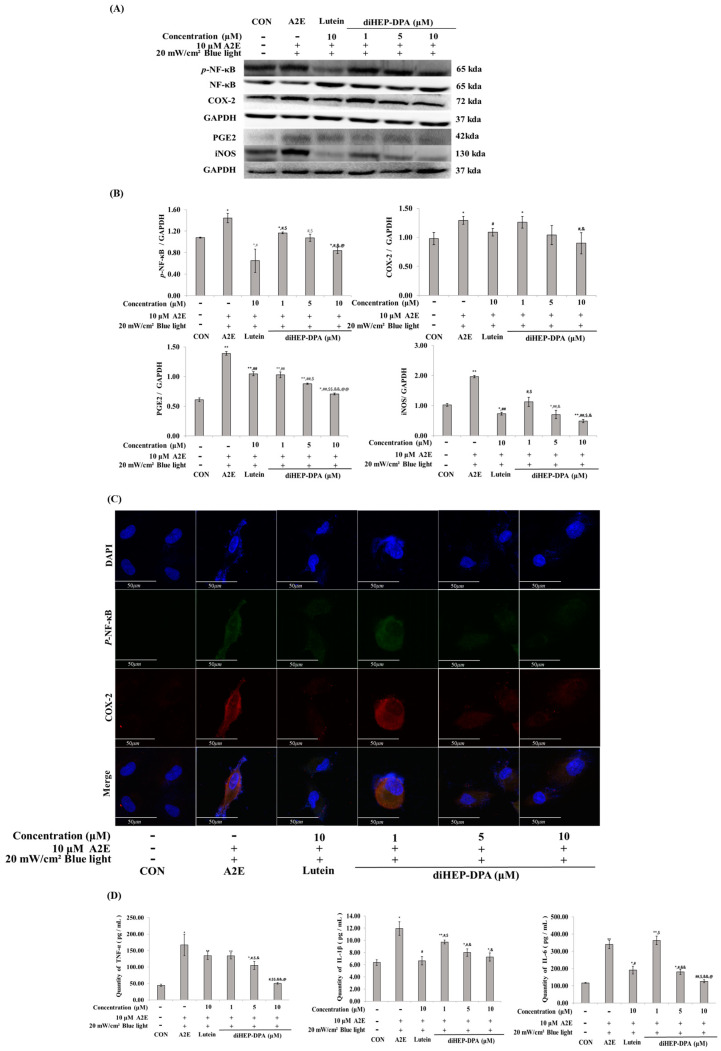
diHEP-DPA decreases inflammatory and pro-inflammatory cytokines induced by blue light in A2E-laden retinal pigment epithelium (ARPE-19) cells. (**A**) Western blot analysis indicating the expression of *p*-NF-κB, NF-κB, COX-2, PGE2, iNOS, and GAPDH. (**B**) Quantifications were approximated using densitometry (Image J software version 1.8.0), and results were normalized to GAPDH. (**C**) Immunofluorescence images showing qualitative expression of P-NF-κB (green), COX-2 (red). Cells were counterstained with DAPI (blue) and visualized by confocal microscopy. Scale bor, 50 µm. Magnifiaction ×400. (**D**) Quantitative analysis of TNF-α, IL-1β, and IL-6 cytokines with an ELISA kit. The values are expressed as the mean ± S.D. (n = 3) of three individual experiments. “*” *p* < 0.05 vs. CON; “**” *p* < 0.001 vs. CON; “#” *p* < 0.05 vs. A2E; “##” *p* < 0.001 vs. A2E; “$” *p* < 0.05 vs. Lutein; “$$” *p* < 0.001 vs. Lutein; “&” *p* < 0.05 vs. 1 µm; “&&” *p* < 0.001 vs. 1 µm; “@” *p* < 0.05 vs. 5 µM; “@@” *p* < 0.001 vs. 5 µM.

diHEP-DPA was confirmed to be effective in regulating the inflammatory factors that were increased by A2E and BL. As shown in Figure 5A,B, diHEP-DPA reduced the expression of p-NF-kB, iNOS, COX-2, and PGE2, which was increased by A2E and BL, in a concentration-dependent manner. In addition, diHEP-DPA decreased the production of proinflammatory cytokines such as TNF-α, IL-6, and IL1-1β in a concentration-dependent manner.

### 3.5. diHEP-DPA Regulated the Oxidative Stress and Carbonyl Stress Induced by A2E and BL Exposure in ARPE-19 Cells

**Figure 6:**
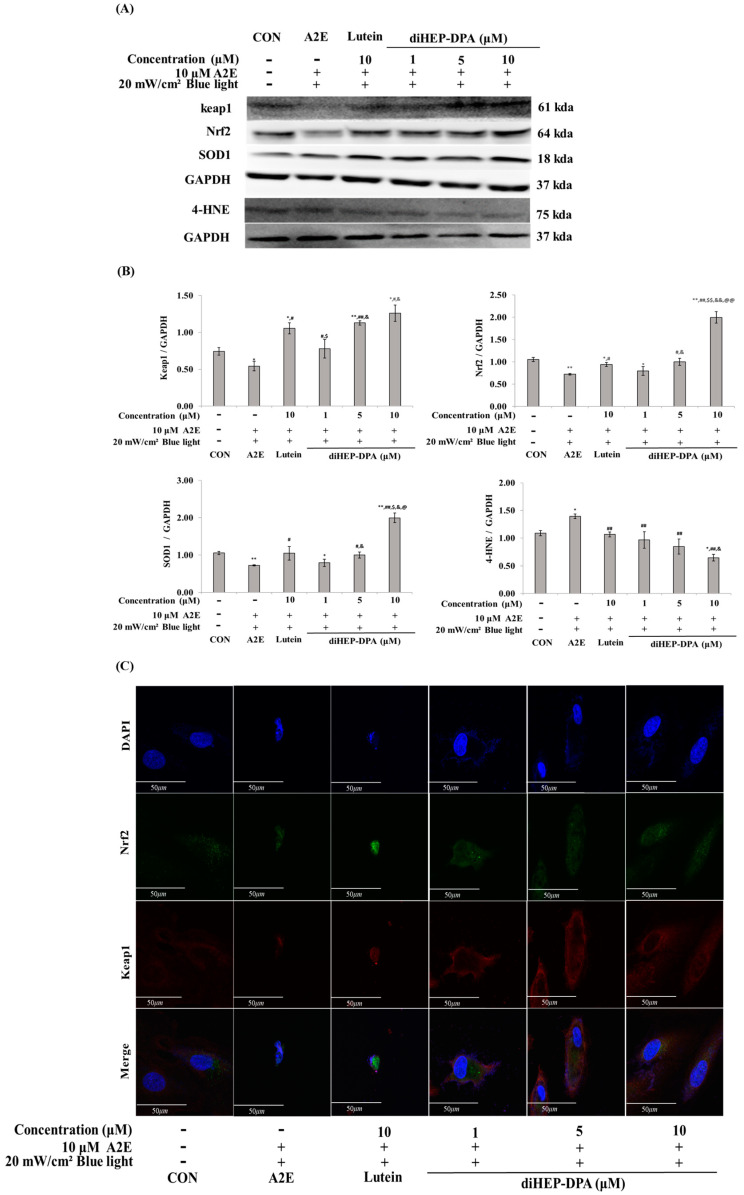
diHEP-DPA decreases oxidative stress and carbonyl stress induced by blue light in A2E-laden retinal pigment epithelium (ARPE-19) cells. (**A**) Western blot analysis indicating the expression of Keap1, Nrf2, SOD1, 4-HNE, and GAPDH. (**B**) Quantifications were approximated using densitometry (Image J software version 1.8.0), and results were normalized to GAPDH. (**C**) Immunofluorescence images showing qualitative expression of Nrf2 (green) and Keap1 (red). Cells were counterstained with DAPI (blue) and visualized by confocal microscopy. Scale bor, 50 µm. Magnifiaction ×400. The values are expressed as the mean ± S.D. (n = 3) of three individual experiments. “*” *p* < 0.05 vs. CON; “**” *p* < 0.001 vs. CON; “#” *p* < 0.05 vs. A2E; “##” *p* < 0.001 vs. A2E; “$” *p* < 0.05 vs. Lutein; “$$” *p* < 0.001 vs. Lutein; “&” *p* < 0.05 vs. 1 µm; “&&” *p* < 0.001 vs. 1 µm; “@” *p* < 0.05 vs. 5 µM; “@@” *p* < 0.001 vs. 5 µM.

To further confirm that apoptosis and inflammatory responses caused by exposure to A2E and BL are related to oxidative/carbonyl stress, proteins typically expressed during oxidative/carbonyl stress were selected and their expression patterns were confirmed. diHEP-DPA increased the expression levels of Keap-1, NRF-2, and SOD1, which were reduced by A2E and BL, in a concentration-dependent manner, and the efficacy of the control group treated with 10 µM lutein and the group treated with 5 µM diHEP-DPA were similar. In the case of 4-HNE, the expression was increased by A2E and BL treatment, and diHEP-DPA showed a concentration-dependent expression pattern at concentrations up to 10 µM (Figure 6A,B). As shown in Figure 6C, the immunofluorescence assay confirmed that diHEP-DPA induced the expression of Kaep-1 and NRF-2 in a concentration-dependent manner.

## 4. Discussion

In the present study, we showed that the treatment of retinal oxide (A2E) with BL caused cytotoxicity in RPE cells. Lutein and diHEP-DPA regulated the apoptosis and inflammatory responses induced by A2E and BL. BL has been reported to cause macular degeneration and cataracts by damaging the eye cells [40]. In previous studies, cell damage has been induced by A2E with BL treatments. Recently, long-term exposure to BL has been reported to cause excessive oxidative stress in dry age-related macular degeneration (AMD); in a mouse in vivo long-term exposure model, BL weakened the retinal layer, caused apoptosis in retinal cells, and intensified the damage to mitochondria. In particular, mitochondrial function has been reported to deteriorate [40].

It was reported that in a rat model of macular degeneration, a diet rich in DHA and EPA reduced the development of retinal lesions. Furthermore, in a rat model of oxygen-induced retinopathy, increasing tissue levels of ω-3 by diet or genetic manipulation reduced pathological retinal neovascularization. In addition to resolvin, another DHA-derived lipid mediator, 10,17(S)-docosatriene, has been identified in both humans and rodents. DHA is generally present in the eye and respiratory mucosa, and SPMs such as protectin D1 have been reported to be produced in the human respiratory tract as well. Therefore, it can be seen that not only DHA but also DHA derivatives act as autacoids in vivo and have strong anti-inflammatory and immunomodulatory properties.

Thus, we reported that diHEP-DPA, a DHA derivative capable of enzymatic mass production, has anti-apoptotic and anti-inflammatory effects on ARPE-19 cells damaged by A2E and BL at a low concentrations of 10 µM or less.

Lutein is a representative eye health medicine widely sold as an ingredient in medicines and health foods. Lutein, one of the carotenoid pigments in the retina, is widely used clinically worldwide as a substance to prevent retinal degeneration [41], and protective and antioxidant effects have been reported in ARPE-19 cell and mouse models [42].

Therefore, we used lutein as a control and determined that it alleviates ARPE-19 cell damage at a concentration of 10 µM.

Studies on diHEP-DPA’s anticancer and ulcerative colitis effects have been previously reported [36,37]. This is the first study to report the eye protective effect of diHEP-DPA. When RPE cells are treated with A2E at an appropriate concentration and irradiated with BL for a certain period, A2E is oxidized to oxi-A2E, which accumulates within the cells, causing cytotoxicity and apoptosis. Retinal epithelial cells are usually damaged by fluorescent dyes such as lipofuscin. The photoreceptor cells die sequentially after cell death [43]. Therefore, we attempted to explain how new lipid autacoids, such as SPMs, protect RPE cells against apoptosis caused by BL. As shown in Figure 1, lutein and diHEP-DPA were shown to alleviate cytotoxicity caused by A2E and BL at the 10 µM level; hence, experiments were conducted at concentrations below 10 µM (Figure 1).

Janet et al. reported that BL exposure causes A2E oxidation and generates reactive oxygen species (ROS), including hydrogen peroxide and superoxide anion [44]. ROS are known to cause oxidative stress and apoptosis [45], and oxidative stress and inflammatory responses are associated with the apoptosis of RPE cells [46].

MAPKs have been implicated in many human pathologies, including neurodegenerative diseases (e.g., Alzheimer’s disease, Parkinson’s disease, and amyotrophic lateral sclerosis), diabetes, obesity, and various cancers [47]. Considering that MAPKs play a central role in most cell signaling systems, changes in the expression and function of various MAPK signaling intermediates have also been reported in RPE cells. It is well known that MAPKs have various effects on cell differentiation. ERK1/2 is associated with cell proliferation and differentiation, and p38 is known to be closely related to inflammation, apotosis, and stress responses in addition to cell proliferation and differentiation. JNK is also associated with apoptosis, cell proliferation, and cell differentiation [48].

In the present study, we confirmed that A2E and BL activate p38 and JNK and are consequently related to apoptotic/inflammatory responses.

Ultraviolet rays are a representative factor affecting eye diseases, and in relation to MAPKs, it has been reported to increase the expression of ERK1/2, JNK, and p38 in RPE cells [49]. Representative substances that regulate MAPKs activity have been reported, including resveratrol derived from grapes [50]. Numerous studies have reported that bioactive substances other than resveratrol regulate MAPK signaling. However, we are the first to report a study on MAPK-mediated apoptosis and inflammation of SPMs, such as diHEP-DPA, by A2E and BL in ARPE-19 cells.

Cigarette smoke and Cd have also been reported to affect RPE cells, and autophagic cell death has been reported to be induced by the activation of ERK, JNK, and p38 [51]. Tsao et al. reported that when RPE cells were treated with hydrogen peroxide, ERK1/2 was not involved in cell death; however, cell death was affected by JNK and p38 activation [52]. Ryter et al. reported that apoptosis occurs through continuous activation of ERK1/2 [53]. In our study, treatment with A2E and BL induced JNK and p38 activation, similar to the results of Tsao et al., in which diHEP-DPA downregulated JNK and p38 activation induced by A2E and BL (Figure 4).

Bcl-xL and BCL-2 contribute to the inhibition and regulation of mitochondrial apoptosis. In particular, overexpression of BcL regulates cytochrome c release from mitochondria and caspase activity [54], and diHEP-DPA increases the expression of Bcl-xL and Bcl-2 by approximately a factor of 1.5 times more than lutein. Additionally, the expression of Bad and Bim, pro-apoptotic factors, was increased by A2E and BL treatment, respectively, and was suppressed by lutein and diHEP-DPA, which is consistent with the report that factors such as Bad form a dimer with Bcl-xL. These results indicate that diHEP-DPA inhibited RPE cell death by regulating the apoptotic pathway (Figure 3).

Retinal cell damage due to visible light exposure occurs via type I (free radical) and type II (oxygen-dependent) mechanisms. Apoptosis is induced by a type II mechanism [55]. Exposure of cells to BL generates ROS, which damage mitochondrial DNA and cell structure and cause RPE cell death [56,57].

In the present study, we found that A2E and BL exposure induces oxidative stress in ARPE-19 cells, affecting the Keap1/NRF2 pathway, and diHEP-DPA regulates Keap1/NRF2 and SOD1 to alleviate apoptosis and inflammation caused by oxidative stress (Figure 6).

Under oxidative stress, cholesterol is converted to oxysterol, another lipid compound vulnerable to ROS attack. Representative substances include 7-keto-cholesterol, 7β-hydroxy-cholesterol, 5α,6α- and 5β,6β-epoxy- cholesterol, and cholestan-3β,5α,6β-triol. These oxysterols have been reported to cause eye diseases (e.g., macular degeneration [58] and dry eye [59]).

Continuous exposure to oxidative stress in RPE cells can damage cellular organelles, such as mitochondria [60], which ultimately causes the worsening of eye diseases, such as macular degeneration. Therefore, oxidative stress-induced RPE damage is considered a key pathological factor.

Microglial activation induced by light exposure causes an inflammatory response, which has been reported to be related to the development of macular degeneration [61]. In particular, it has been reported that reactive microglia accumulate in the subretinal space and release pro-inflammatory cytokines such as TNF-α, IL-1β, and IL-6 [62].

Many reports on the molecular mechanisms underlying the link between oxidative stress and inflammation in RPE cells have not been confirmed. However, Yang et al. reported that 4-HNE induces the production of IL-6, IL-1, and TNF-α through HSP70 outflow from RPE cells [63]. In our study, we confirmed that when RPE cells were treated with A2E and BL, 4-HNE expression was induced by oxidative stress, and diHEP-DPA alleviated this effect (Figure 6). This result is related to the data in Figure 5, in which the cytokines (TNF-α, IL-1β, and IL-6) increased by A2E and BL treatment were downregulated by diHEP-DPA.

Taken together, diHEP-DPA treatment alleviated the apoptosis and inflammatory responses due to oxidative and carbonyl stresses induced by A2E and BL treatment in ARPE-19 cells in a concentration-dependent manner at concentrations below 10 µM.

## 5. Conclusions

We demonstrated that diHEP-DPA, obtained by the enzymatic reaction of DHA, can regulate apoptosis and inflammatory responses induced by A2E and BL. It was confirmed that diHEP-DPA regulates the expression of MAPK-related proteins related to apoptosis in RPE cells and is involved in apoptosis and inflammatory responses caused by oxidative and carbonyl stresses. For the development of diHEP-DPA as a treatment material for macular degeneration in the future, in vivo and pathological analyses are necessary to apply diHEP-DPA as an eye drop preparation.

In this study, we showed that SPMs produced in human cells have limitations in treating diseases caused by exposure to A2E and BL, and that substances such as diHEP-DPA are sufficient as potential treatments for eye diseases.

## Data Availability

Should any raw data files be needed; they are available from the corresponding author upon reasonable request.
